# Esophageal achalasia: An unusual reason for lung abscess

**DOI:** 10.1002/jgf2.518

**Published:** 2022-01-14

**Authors:** Seiji Shiota, Ryoko Kuribayashi, Rie Utsunomiya, Eishi Miyazaki

**Affiliations:** ^1^ Department of General Medicine Oita University Faculty of Medicine Yufu Oita Japan

**Keywords:** esophageal achalasia, lung abscess

## Abstract

Our case serves as a reminder that clinicians should pay attention to the presence of esophageal diseases in patients with lung abscesses. The esophagus should be evaluated during pulmonary CT imaging.

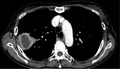

A 62‐year‐old woman was referred to our hospital for assessment of low‐grade fever and cough lasting 6 days and 4 months, respectively. She had lost 5 kg during the previous 5 months. She had a history of dysphagia for the past 4 years and had been diagnosed with bronchitis 5 years earlier. She had no history of bronchial asthma or diabetes mellitus, although she had a 20‐pack‐year smoking history. On admission to our hospital, her vital signs were as follows: temperature, 37.3°C; blood pressure, 96/65 mmHg; heart rate, 102 beats/min; respiratory rate, 14 breaths/min; and peripheral oxygen saturation, 98% on room air. She was fully oriented and responsive. Physical examination was normal. Laboratory findings on admission showed a white blood cell count of 13,190/μl and C‐reactive protein (CRP) level of 4.1 mg/dl. All other laboratory findings were unremarkable. Computed tomography (CT) of the chest showed a 50 mm abscess with air‐fluid levels in the right upper lung lobe (Figure 1a,b). Antibiotic treatment was commenced for the lung abscess, with treatment success. CT also showed dilation of the esophagus, suggestive of esophageal achalasia. Barium swallow showed dilatation of the esophagus with eccentric tapering (Figure 2). High‐resolution manometry confirmed the diagnosis of esophageal achalasia. Pulmonary function tests indicated obstructive airway defects. After antibiotic treatment of the lung abscess, per‐oral endoscopic myotomy (POEM) for esophageal achalasia was performed. At follow‐up 12 months after surgery, she did not show any relapse of pneumonia or lung abscess. Follow‐up CT showed resolution of the lung abscess (Figure 1c,d), although pulmonary function tests still showed obstructive airway defects. The patient provided written informed consent for publication.

**FIGURE 1:**
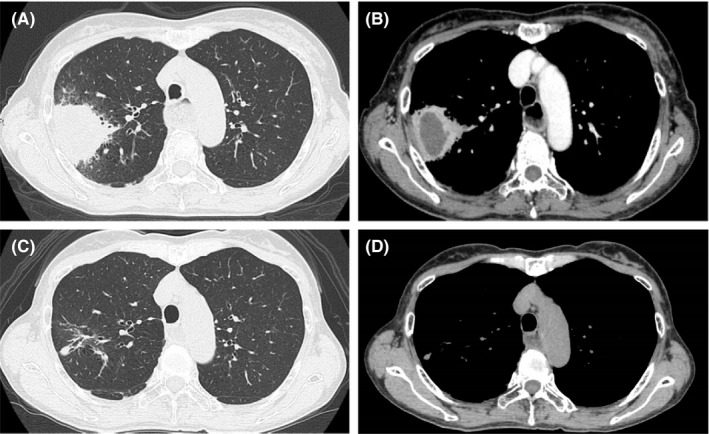
(A,B). Computed tomography (CT) of the chest showed a 50 mm abscess with air‐fluid levels in the right upper lung lobe. CT also showed dilatation of the esophagus, suggestive of esophageal achalasia. (C,D) Follow‐up CT showed resolution of the lung abscess

**FIGURE 2:**
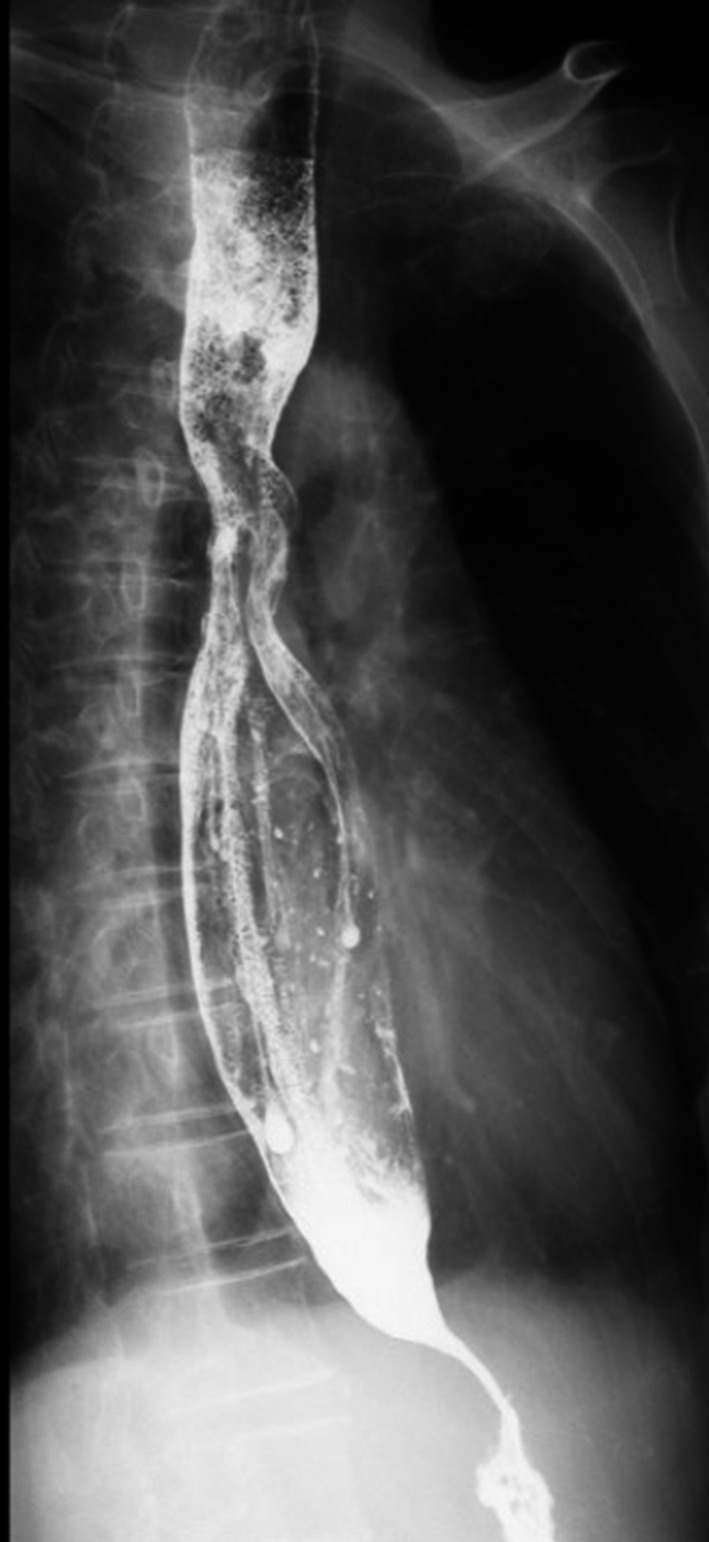
Barium swallow showed the dilated esophagus with eccentric tapering

Esophageal achalasia is a primary esophageal motor disorder of unknown etiology characterized manometrically by insufficient lower esophageal sphincter relaxation and loss of esophageal peristalsis.^1^ The most common symptoms are dysphagia for both solids and liquids, regurgitation, and chest pain.^1^ In addition to Heller myotomy and esophagectomy,^2^ POEM has been established as a principal endoscopic therapeutic procedure in recent years.^2^, ^3^ Esophageal achalasia induces lung abnormalities due to dilatation of the esophagus and recurrent microaspiration.^4^ A previous report showed that 50% of patients with esophagus achalasia had evidence of lung lesions such as tracheo‐bronchial compression by the dilated esophagus, ground‐glass opacities, nodules, fibrosis, air trapping, consolidation, and bronchiectasis on CT.^4^ Furthermore, esophageal achalasia causes aspiration pneumonia, which can be lead to lung abscess.^1^ In addition, 20% of patients had either restrictive or obstructive airway diseases.^4^ Treatment of esophageal achalasia provided relief of dysphagia, respiratory symptoms, and radiological and functional changes in the previous cases.^5^ Our case experienced relief of symptoms and the radiological lesion, but with no improvement in pulmonary function test results after POEM. Longitudinal follow‐up after treatment is necessary to elucidate the changes induced by esophageal achalasia.

Our case serves as a reminder that clinicians should pay attention to the presence of esophageal diseases in patients with lung abscesses. The esophagus should be evaluated during pulmonary CT imaging.

## CONFLICTS OF INTERESTS

The authors have stated explicitly that there are no conflicts of interest in connection with this article.
